# Malicious UAV Detection Using Integrated Audio and Visual Features for Public Safety Applications

**DOI:** 10.3390/s20143923

**Published:** 2020-07-15

**Authors:** Sonain Jamil, Fawad, MuhibUr Rahman, Amin Ullah, Salman Badnava, Masoud Forsat, Seyed Sajad Mirjavadi

**Affiliations:** 1 ACTSENA Research Group, Telecommunication Engineering Department, University of Engineering and Technology, Taxila, Punjab 47050, Pakistan; 16-te-16@students.uettaxila.edu.pk (S.J.); engr.fawad@students.uettaxila.edu.pk (F.); 2 Department of Electrical Engineering, Polytechnique Montreal, Montreal, QC H3T 1J4, Canada; 3 College of Engineering & Computer Science (CECS), Center for Research in Computer Vision Lab (CRCV Lab), University of Central Florida (UCF), Orlando, FL 32816, USA; amin.ullah@uettaxila.edu.pk; 4 Department of Computer Science and Engineering, College of Engineering, Qatar University, P.O. Box Doha 2713, Qatar; 5 Department of Mechanical and Industrial Engineering, College of Engineering, Qatar University, P.O. Box Doha 2713, Qatar; mf1904885@qu.edu.qa (M.F.); smir512@aucklanduni.ac.nz (S.S.M.)

**Keywords:** AlexNet, feature extraction, localization, public safety, malicious drones, surveillance

## Abstract

Unmanned aerial vehicles (UAVs) have become popular in surveillance, security, and remote monitoring. However, they also pose serious security threats to public privacy. The timely detection of a malicious drone is currently an open research issue for security provisioning companies. Recently, the problem has been addressed by a plethora of schemes. However, each plan has a limitation, such as extreme weather conditions and huge dataset requirements. In this paper, we propose a novel framework consisting of the hybrid handcrafted and deep feature to detect and localize malicious drones from their sound and image information. The respective datasets include sounds and occluded images of birds, airplanes, and thunderstorms, with variations in resolution and illumination. Various kernels of the support vector machine (SVM) are applied to classify the features. Experimental results validate the improved performance of the proposed scheme compared to other related methods.

## 1. Introduction

Mini drones, also known as unmanned aerial vehicles (UAVs), have played a vital role in the development of smart cities. The UAVs have numerous industrial and agricultural applications. The high-resolution images collected through UAVs help in various monitoring applications of the cement industry [1]. Drones are helpful in the irrigation [2] and carrying chemical pesticides or fertilizers to spray on plants [3]. So-called foggy drones use thermal cameras to scan the roads and avoid accidents in foggy weather [4]. The UAVs can operate as mobile base transceiver stations (BTS) to facilitate the surge traffic demands during disasters [5,6]. In smart cities, drones resolve cybersecurity issues [7]. UAVs also help in the navigation and positioning of military targets during war [8]. 

**Figure 1:**
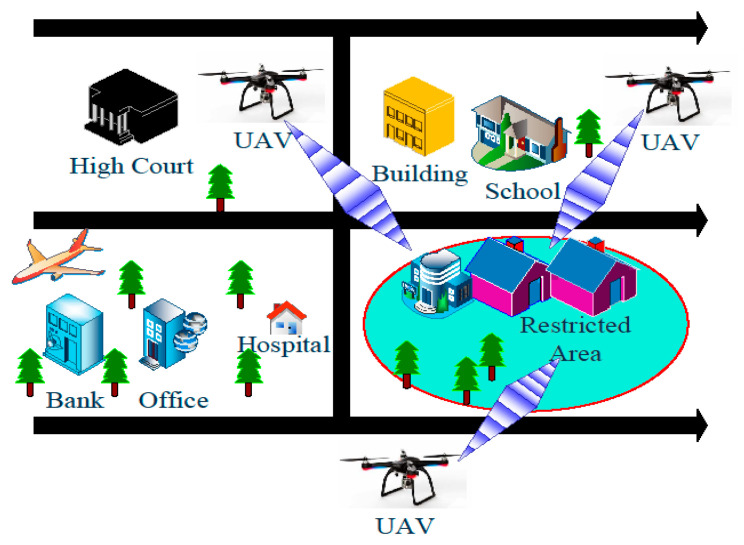
Intrusion of malicious drones.

Malicious UAVs are those which either carry restricted explosive payload or collect audiovisual data from restricted private geographic territory. Moreover, a UAV can be considered malicious when it loses control and enters the nonflying zone [9]. The low-altitude flight of a malicious drone enables it to violate the security measures of a restricted zone, as shown in Figure 1. Restricted areas protect sensitive locations, such as prisons and nuclear facilities. The official definition of such a restricted area is “an airspace of defined dimensions above the land areas or territorial waters of a State within which the flight of aircraft is restricted under certain specified conditions”.

There is a need for a technology that can detect and disarm such malicious UAVs in a timely manner. Recently, various techniques for UAV detection have been reported in the literature, relying on audio, video, thermal, and radio frequency (RF) signals [10]. Each scheme has its own advantages and limitations. The video- and thermal-based detection techniques fail in adverse weather conditions. The sound of a UAV’s motor fan and its images are useful to differentiate the amateur UAV from other objects. The audio-based detectors are cost-effective as they require only an array of microphones to capture the sounds and classify them in their respective class. However, environmental noise can degrade the performance of sound-based detection [11].

We propose a machine-learning-influenced audio- and vision-based UAV detection method. The proposed scheme is capable of detecting UAVs with higher accuracy, even in a noisy environment. The proposed hybrid method consists of acoustic and image processing algorithms for the precise detection of amateur drones [10,11]. The classification accuracy obtained using handcrafted and deep neural network is compared with the proposed framework. Various handcrafted feature extraction methods for image description, such as Local Binary Pattern (LBP) [12], Histogram of Oriented Gradient (HOG) [13], Locally Encoded Transform Feature Histogram (LETRIST) [14], Gray Level Co-occurrence Matrix (GLCM) [15], Completed Joint-scale Local Binary Pattern (CJLBP) [16], Local Tetra Pattern (LTrP) [17], and Non-Redundant Local Binary Pattern (NRLBP) [18], have been employed to detect objects based on their texture. Moreover, several handcrafted feature extraction methods for audio have been proposed, such as Linear Predictive Cepstral Coefficients (LPCC) [19], and Mel Frequency Cepstral Coefficients (MFCC) [20]. The deep neural network (DNN) models such as: AlexNet [21], ResNet-50 [22], VGG-19 [23], Inceptionv3 [24], and GoogLeNet [25] have also been utilized for image feature extraction. The support vector machine (SVM), along with various kernels, have been employed to classify the extracted feature vectors. The proposed scheme is cost-effective as well as highly accurate, even with a small dataset. The proposed scheme integrates the handcrafted sound descriptor with deep features extracted from the image to detect the malicious drone. This hybrid method has provided better accuracy even in adverse weather conditions [11].

## 2. Related Work

UAVs can efficiently be detected via several intrinsic signals, which are thermal images, the sound of the UAV’s motors, and radio frequency (RF) radar [10]. In [26], the authors achieved 81% UAV detection accuracy by extracting features from the input array of cameras and microphones. In [27], a pseudorandom sequence of binary values was presented to detect drones. The results show that the pseudorandom sequence can only detect UAVs within the 100 m range for the 2 GHz band. The technique in [28] proposed a radar that operates at 35 GHz frequency-modulated continuous-waves (FMCW) equipped with fixed antennas. The results show that their estimated velocities efficiently detected UAVs. This system can be made more efficient by employing circularly polarized antennas. In [29,30], deep belief network (DBN) along with convolutional neural networks (CNNs) were reported. The DBN accuracy depends on channel conditions; moreover, they require a huge dataset for accurate detection. In [31,32], texture descriptors were developed that can classify surfaces into their respective classes even in the presence of geometric and photometric variations. In [33], a tracker was developed by employing the handcrafted descriptors proposed in [31,32].

Furthermore, the authors in [34] measured the radio signal in cellular networks using logistic regression and decision tree to detect drones. The accuracy of these models is reduced when drones are flying at lower heights. Similarly, in [35], plotted image machine learning (PIL) and K-nearest neighbors (KNN) were developed for acoustic-based drone detection in the real-time scenario. The simulation results show that PIL is 22% more accurate than KNN, while KNN is less complicated than PIL. These approaches require a massive amount of data for better performance. 

In [28], the authors present a limited-dataset-dependent algorithm for correlation-based sound detection. The method is cost-effective, but it is not suitable for real-time applications. In [36,37], a video-based mechanism was developed for robust detection of drones. In this scheme, the system is equipped with two cameras with day and night vision sensors. The short-wave infrared (SWIR) cameras along with high-resolution visual-optical (VIS) cameras were included with the above system. Still, it failed to bring improvement in accuracy. The mechanism in [37] failed to work properly in strong wind. In [38], Hidden Markov Model (HMM) was used to detect UAVs using acoustic sensors. This model also has limitations, as it gives a poor performance for a small amount of training data due to the complexity of classifiers. There is no such scheme, according to the authors’ knowledge, that can detect UAVs accurately using a small amount of training data and machine learning algorithms. This paper contributes to detecting UAVs through a hybrid approach; the first part is related to the detection of UAVs by their sound, while the second part consists of UAV detection and localization using images. 

## 3. UAV Detection Methodology

UAVs have specific acoustic features that are different than other sounds in the surrounding environment. The sounds play a vital role in UAV detection if appropriate features are extracted and classified. On the other hand, UAVs are very different in shape than the surrounding object, so the image can be a piece of information that is useful to detect UAVs. The image features are extracted by a convolutional neural network (CNN) like AlexNet, and then the extracted features are classified using some efficient classifier.

**Figure 2:**
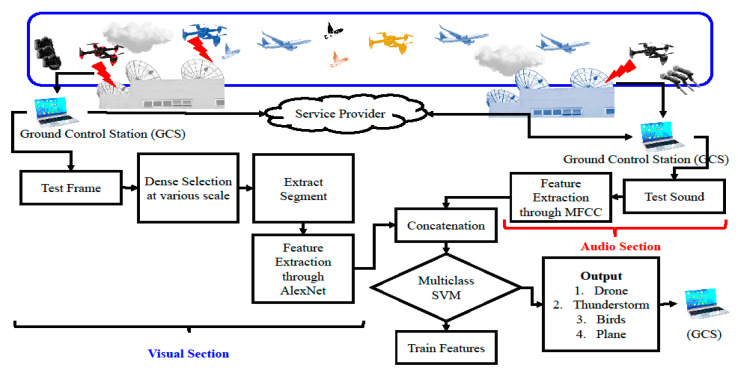
Audio and visual feature-based UAV detection system model.

The proposed malicious UAV detection model depends on the audio and images collected within the restricted zone, as shown in Figure 2. The arrays of microphones and high-resolution cameras capture the audio and video within the restricted zone. First, the ground control stations (GCS) collect the audio and visual information from the respective array of sensors. In the second stage, features are extracted from the audio and visual information through a specified descriptor. In the third step, the extracted features are classified using a trained classifier. In this paper, we have used a machine learning technique to classify the audio and image features extracted through the MFCC and AlexNet model, respectively. The SVM with various kernels is used as a classifier.

### 3.1. Audio Feature Extraction

**Figure 3:**
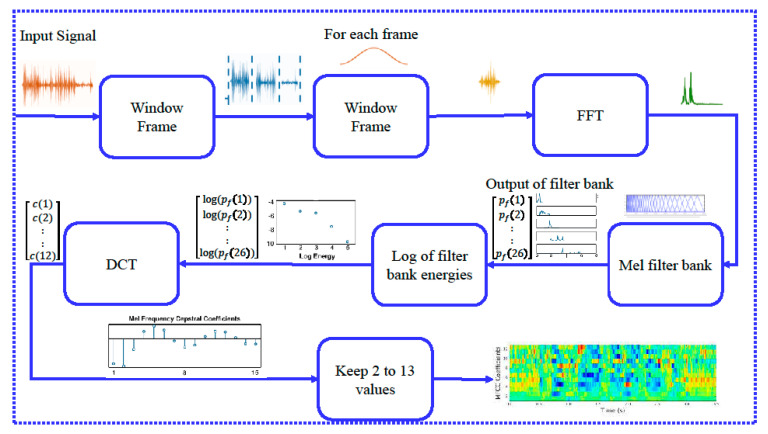
Mel Frequency Cepstrum Coefficients (MFCC) computational steps.

The audio features are extracted through Mel Frequency Cepstrum Coefficients (MFCC) descriptor. In MFCC, the frequency axis is enveloped with Mel frequencies [20]. Firstly, the pre-emphasis and windowing filter is applied to audio. Secondly, the Fast Fourier transform is applied over the filtered sound signals, following the Mel filter banks. In the third stage, the log of the filter bank energies is calculated. Finally, the discrete cosine transform (DCT) is applied, and the resultant values between 2 and 13 are preserved, while the rest are discarded. The output of DCT is MFCCs, and all the steps, as mentioned earlier, are illustrated in Figure 3. The frequency in hertz (Hz) is converted into the Mel frequency scale through the following Equation (1).

(1) $$mel\left(f\right)=2595log10\left(\frac{f}{700}+1\right)$$

The symbol *mel* in Equation (1) represents the frequency in the Mel scale, while the symbol *f* represents the frequency in hertz. The Mel spectrum is the result of the log of filter banks. The DCT is applied on the Mel spectrum to get Mel cepstrum coefficients, as shown in Equation (2).

(2) $$c\left(n\right)=\overset{M-1}{\underset{m=0}{\sum }}\left(logD\left(m\right)\cos(\frac{\pi n\left(k-0.5\right)}{M})\right);n=0,1,\ldots ,C-1:0\leq k\leq M-1.$$

The function *c*(*n*) in Equation (2) represents the MFCC coefficients, while the symbol *C* is the size of MFCC coefficients. The function *D*(*m*) denotes the Mel magnitude spectrum. The Mel magnitude spectrum is the product of the magnitude spectrum and the triangular Mel weighting filters. The *m* is the *m-*th triangular filter coefficient. The variable *k* in Equation (2) denotes the index of the sample, while *M* represents the total number of samples. 

### 3.2. Visual Feature Extraction

**Figure 4:**
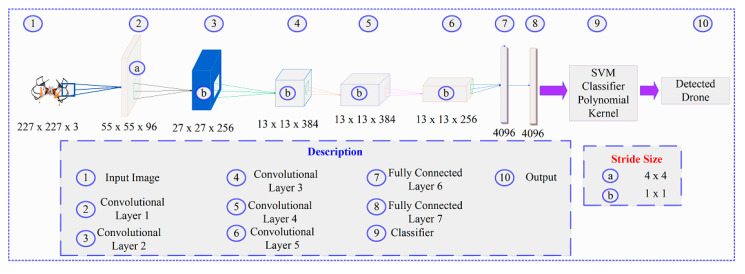
Drone detection through AlexNet.

AlexNet is used to extract features for the image. It has 25 layers: one input layer, one output layer, and 23 hidden layers. The hidden layers consist of five convolutional layers, three max-pooling layers, seven rectified linear unit (ReLU) layers, three fully connected layers, two cross-channel normalization layers, two dropout layers, and one softmax layer. The feature extraction using AlexNet is shown in Figure 4. The size of the input image is 227 × 227 × 3 at the input layer of AlexNet. This input is fed into the first convolutional (C1) layer, which has 96 kernels, and stride size in it is 4 × 4. The remaining convolutional layers are cascaded to C1 with the stride size of 1 × 1.

### 3.3. Support Vector Machine (SVM)

**Figure 5:**
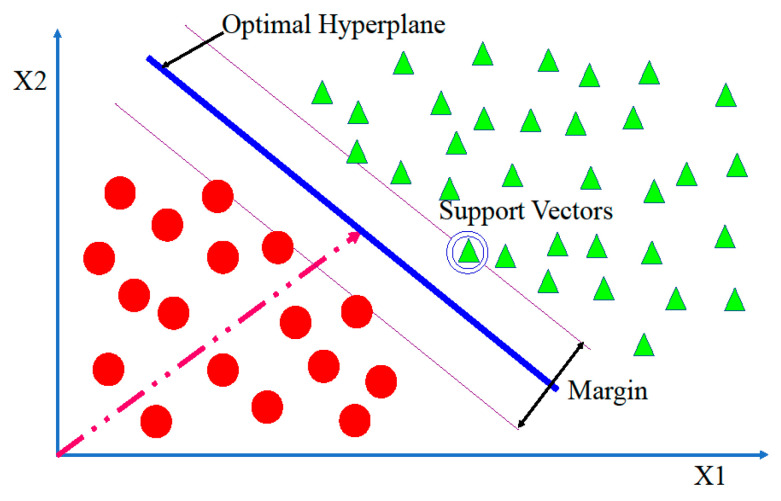
Support vector machine (SVM).

In this paper, SVM is used to classify the extracted features. The SVM set its hyperplane based on positive and negative training feature set to minimize the classification error. The hyperplane adjusts itself in such a way that it reduces the classification error, as shown in Figure 5. The hyperparameters of SVM that are linear, Gaussian, and polynomial kernel have been used to classify features. SVM chooses the ideal choice limit contingent on the most extreme edge, which ideally isolates the information focuses. Grouping mistake proportion is limited as edge increments, and thus increases the edge, which results in the least mistakes [39]. The preparation guides closer toward the ideal isolating hyperplane are the support vectors [40]. This can be written as in Equation (3).

(3) $$\omega^{T}x+\beta =\pm 1$$

where *β* denotes the bias, while the symbols *x* and *ω* are vectors representing the input and its weight, respectively. When the extracted features have a higher dimensionality, then the learning process selects those variables having a higher interclass variation. This technique is generally known as a bit trap [41]. The favorable principle position of SVM kernels is their capacity to work in any measurements with no extra calculations and multifaceted nature. SVM can perform better even for the noisy high-dimensional feature vectors. This persuades us to choose SVM as a classifier. For SVM grouping precision, selecting a suitable part plays an essential job. We compared the classification accuracy of SVM with its linear, Gaussian, and polynomial kernel types. Equation (4) is for linear kernel. For the polynomial kernel, Equation (5) is used.

(4) $$K\left(x_{i},x_{j}\right)=x_{i}^{T}x_{j}$$

(5) $$K\left(x_{i},x_{j}\right)={\left(1+x_{i}^{T}x_{j}\right)}^{p}$$

Here the symbols $x_{i}$ and $x_{j}$ are vectors’ dot product and are plotted in the space of dimension *p*. The following equation, Equation (6), is used for the Gaussian kernel.

(6) $$K\left(x_{i},x_{j}\right)=\frac{\exp\left(-\|x_{i}-x_{j}\|{}^{2}\right)}{2\sigma^{2}}$$

where $\left\|x_{i}-x_{j}\right\|$ is used to calculate the euclidean distance of two different samples. The width of the Gaussian kernel can be controlled by changing the value of the variance σ.

SVM is trained using features extracted from AlexNet for the visual dataset and MFCC-extracted features in the case of the audio dataset. The hyperparameters for SVM training are kernels.

## 4. Experimental Results

In this section, we evaluated UAV detection using integrated audio and visual features by using audio and image datasets. The dataset is classified by implementing an SVM classifier. Malicious UAVs are localized by implementing handcrafted descriptors like HOG, LBP, CJLBP, LTrP, GLCM, NRLBP, and LETRIST as well as deep neural networks like AlexNet, inceptionv3, VGG-19, resNet50, and GoogleNet. While using an acoustic dataset, malicious UAVs can be detected by implementing MFCC, LPCC, and ZCR in MATLAB. All the experiments were run on a computer with an Intel(R) Core i7 processor (3.6 GHz) and 16 GB DDR4 RAM. CyberpowerPC, Gamer Supreme Liquid Cool, SLC8260A2. 

### 4.1. Image Dataset Description

**Figure 6:**
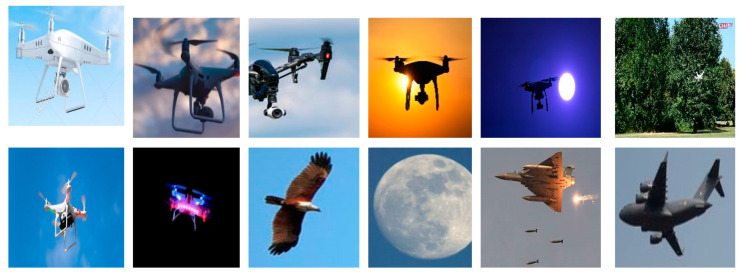
Dataset images.

We implemented the proposed method with the dataset of 506 images. Three hundred fifty images were used for training, while 156 images were used for the test. The images were selected randomly with the ratio of 70% for training and 30% for testing. The dataset consists of five classes of the images that are birds, airplanes, kites, balloons, and drones. The flight scenarios of the dataset are low altitude, high altitude, bad weather, bad visibility, clear weather, and noisy environment. The images of the dataset have variations in their resolution, scale, orientation, and illumination. Moreover, drone images also have environmental occlusions. Several pictures from the dataset are presented in Figure 6. 

### 4.2. Audio Dataset Description

**Figure 7:**
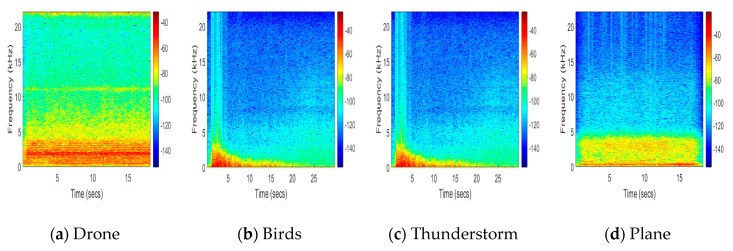
Spectrograms of audio samples. (**a**) Drone; (**b**) Bird; (**c**) Thunderstorm; (**d**) Plane.

We implemented the proposed method with the dataset of 217 audio samples. One hundred fifty-seven audio samples were used for the training model, and 60 audio samples were used for the test. The audio samples were randomly selected with the ratio of 70% for training and 30% for testing. The dataset contained audio samples of drones, airplanes, birds, and thunderstorms. All the audio samples were different in length. The spectrograms with a sampling frequency of 44 kHz of audio samples of drone, bird, thunderstorm, and plane are shown in Figure 7a–d, respectively. The drone spectrogram contains a red line which means that the drone has specific frequencies, i.e., 2.4 kHz, while this red line is not observed in spectrograms of other audio samples because they have low frequencies as well as high frequencies.

### 4.3. Malicious UAV Detection with Hand-Crafted Descriptors

We used hand-crafted descriptors such as HOG, LBP, CJLBP, NRLBP, GLCM, LTrP, and LETRIST to detect malicious UAVs. We used SVM as a classifier. The implemented code of all handcrafted descriptors is available at [42]. Accuracy of each descriptor with various kernels of SVM has been presented in the Table 1.

### 4.4. UAV Detection with CNNs

**Figure 8:**
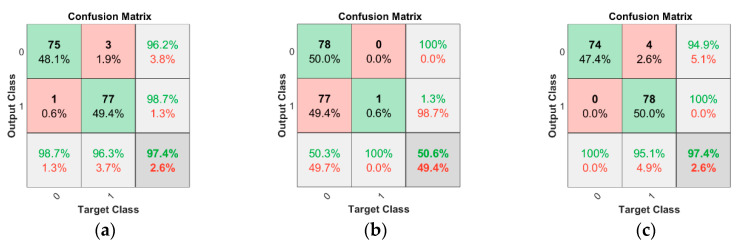
Confusion matrix of AlexNet with different kernels of SVM. (**a**) Linear kernel; (**b**) Gaussian kernel; (**c**) Polynomial kernel.

Results proved that hand-crafted descriptors are not very efficient in malicious UAV detection, as their maximum accuracy is 82.7%. Then, we used CNNs such as AlexNet, inceptionv3, resNet50, GoogleNet, and VGG-19 for the detection of malicious UAVs. The CNN models are used as a descriptor by collecting feature values from the fully connected layer of each respective model. The accuracy, sensitivity, and specificity of all CNNs are shown in Table 2 using different kernels of the SVM classifier. The source codes of all implemented CNNs are available at [43]. The accuracy of AlexNet using the linear or polynomial kernel of the SVM classifier is the greatest among all other CNNs, and it is 97.4%. The confusion matrices of AlexNet using the linear kernel, Gaussian kernel, and polynomial kernel of SVM are shown in Figure 8a–c, respectively.

A confusion matrix is a table that is often used to describe the performance of a classification model (or “classifier”) on a set of test data for which the actual values are known. The diagonal elements of the confusion matrix express the percentage of correct classification, while the other items represent the wrong prediction of the classifier. As the accuracy of AlexNet using the linear and polynomial kernel of SVM is 97.4%, we propose detection of malicious UAVs with AlexNet using the polynomial kernel of SVM because its sensitivity is more significant than the linear kernel and it is more robust by image variations such as resolution, scale, orientation, illumination, and occlusions.

The parameters TP, FP, TN, and FN are true-positive, false-positive, true-negative, and false-negative test samples, respectively. For each threshold, two values are calculated: the true-positive ratio (TPR) and the false-positive ratio (FPR). The TPR is the ratio of TP and the sum of TP and FN. The TPR is known as sensitivity. Equation (7) is used to calculate sensitivity.

(7) $$Sensitivity\;\left(TPR\right)=\frac{TP}{TP+FN}$$

Specificity is another parameter which tells the proportion of correctly identified negative instances. Equation (8) can be used to find specificity.

(8) $$Specificity=\frac{TN}{TN+FP}$$

Overall accuracy and error of classifier is calculated as in Equations (9) and (10) respectively.

(9) $$Accuracy=\frac{TP+TN}{TP+FP+TN+FN}$$

(10) $$Error=\frac{FP+FN}{TP+FP+TN+FN}$$

**Figure 9:**
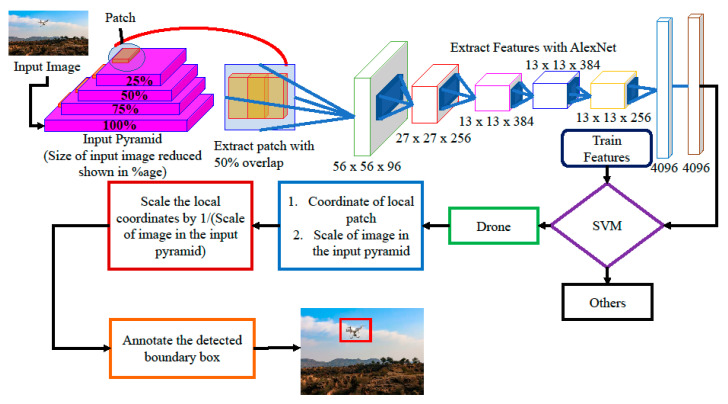
Drone localization steps.

**Figure 10:**
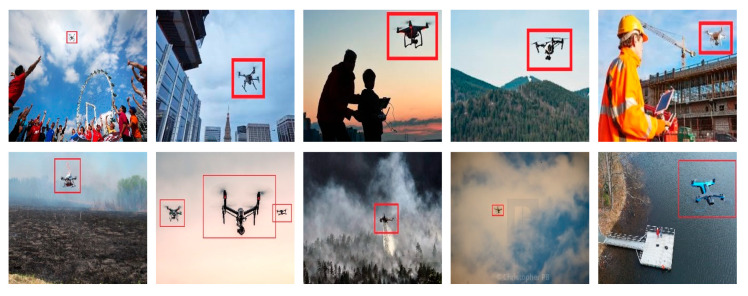
Malicious UAV localization.

As the accuracy of AlexNet is the greatest, we used it for the localization of malicious UAVs in full images. We used training features and training labels of AlexNet that were calculated from the images dataset for localization purposes. Localization procedure is explained in Algorithm 1 and Figure 9. The input image is first scaled into various sizes by creating a scale pyramid, where the fixed size patches are collected from each scale with a 50% overlap. Each local patch is described and classified through the proposed model shown in Figure 9. The size, along with coordinate values of the detector drone, is transformed into the actual image coordinated by the scaling process shown in the figure, and a bounding box annotation is created against those coordinates. Results of localization are shown in Figure 10.
**Algorithm 1.Localization Algorithm**
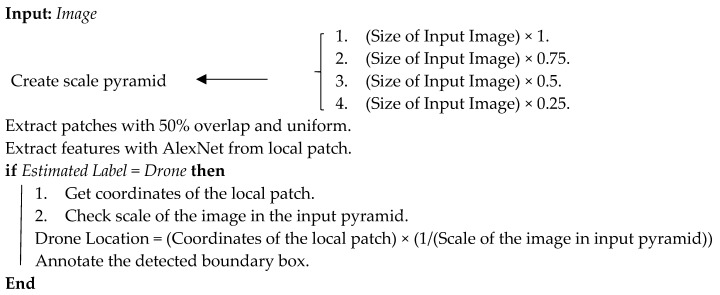


### 4.5. Detection Using Audio

**Figure 11:**
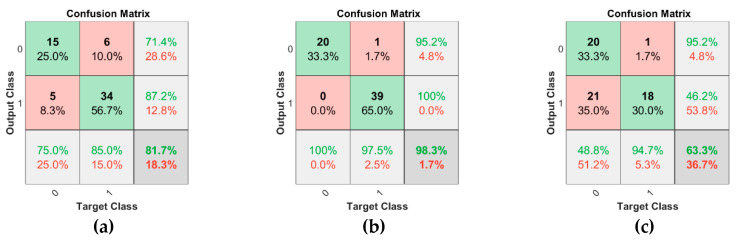
Confusion matrix of MFCC through various kernels of SVM. (**a**) Linear Kernel (**b**) Gaussian Kernel (**c**) Polynomial Kernel.

**Figure 12:**
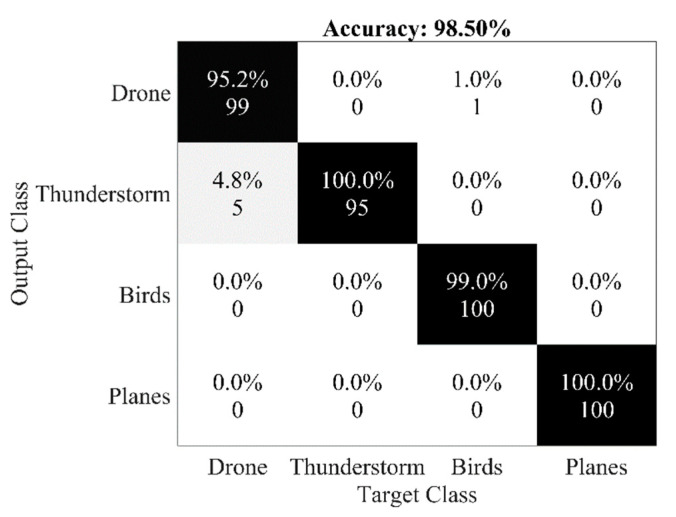
Accuracy of multiclass SVM for combined MFCC and AlexNet features.

We used two descriptors, i.e., LPCC and MFCC, to detect UAVs using audio samples. We used the SVM classifier and calculated accuracy by the confusion matrix in MATLAB. The implemented code is available at [44]. Table 3 shows the accuracy, sensitivity, and specificity of all the descriptors using different kernels of SVM. MFCC proved to be very effective in UAV detection with a Gaussian kernel of SVM. This is because its frequency domain characteristics provide better diversity gain. The confusion matrices of MFCC using the linear kernel, Gaussian kernel, and polynomial kernel of SVM are shown in Figure 11a–c, respectively. We also created a combined dataset of images and audio samples [45]. The dataset contains four classes labeled as Drones, Thunder, Birds, and Planes. The dataset contains two sections. The first one is training data, which includes 885 images and audio samples. The second one is testing data, which consists of 400 images and sounds. We combined MFCC features of audio samples and features extracted from AlexNet of images. The combined features are given to multiclass SVM. We observed that the combined approach gives an accuracy of 98.5%. The accuracy of multiclass SVM for this approach is shown in Figure 12, and its source code is available at [46].

### 4.6. Computational Time

The time taken to extract features of one image of size 227 × 227 × 3 through AlexNet is 1.16 s, while the time taken to extract features of one audio sample with MFCC is 0.3 s. The total time taken to train the model for the visual dataset was 16 min, while the total time taken to train the model for the audio dataset was 2 min. The time taken to train the model with a combined dataset was 30 min. The trained model classifies the objects within 2 s.

### 4.7. Comparison with Present Detection Methods

**Figure 13:**
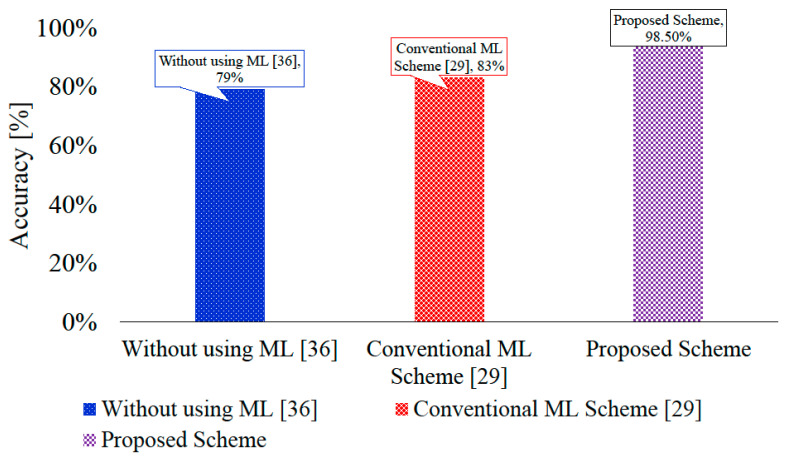
Comparison of proposed UAV detection with conventional scheme and with schemes without using machine learning.

Table 4 shows a comparison of our proposed method with existing drone detection methods. We also compared our work with existing methods to detect drones, i.e., using conventional machine learning and without machine learning, which have detection accuracies of 83% and 79%, respectively. We adopted similar k-fold validation criteria as mentioned in recently published work. We adopted k = 5 for audio, image, and combined datasets. Figure 13 shows that the proposed method achieved almost 98.5% accuracy for drone detection. In the proposed technique, the challenges were low resolution, occlusion, and noisy audio. These challenges are not considered in previous approaches.

## 5. Conclusions

Malicious UAVs have been a challenge for national agencies to consider due to their ability to carry explosive materials. There is a need to detect and localize these UAVs promptly in order to disarm them. For this, a high precision rate model should be used. In this paper, we compared the performance of various hand-crafted descriptors and different CNNs to detect and localize malicious UAVs using a relatively small dataset of images, and we also used MFCC and LPCC to detect malicious UAVs using an audio dataset. We used SVM as a classifier. Our goal was to achieve high accuracy, and the experimental results showed that the accuracy of AlexNet is 97.4% using the polynomial kernel of SVM. The accuracy of MFCC was 98.3% using Gaussian kernel of SVM. Finally, we conclude that AlexNet performed accurately for localization of malicious UAVs, while MFCC had a high precision rate in detecting UAVs based on sound, even in a noisy environment. The combined features of MFCC and AlexNet gives an accuracy of 98.5%. The proposed model can quickly be adopted and deployed by national security agencies to quickly and accurately detect and localize malicious UAVs. This model is cost-effective, as a relatively small dataset is used. In the future, we have a plan to include the RCNN technique and wireless communication in the proposed model.

## Figures and Tables

**Table 1:** Accuracy of the hand-crafted descriptors.

Descriptor	Linear	Gaussian	Polynomial
HOG [13]	82.7%	50.6%	50.6%
LBP [12]	53.8%	59.0%	62.2%
GLCM [15]	74.4%	72.4%	73.1%
CJLBP [16]	75.6%	50.6%	50.0%
NRLBP [18]	50.6%	51.3%	50.0%
LTrP [17]	61.5%	50.6%	50.0%
LETRIST [14]	57.1%	50.6%	50.0%

**Table 2:** Classification results of convolutional neural networks (CNNs) using different kernels of SVM.

**AlexNet [21]**			
**Kernel**	**Accuracy**	**Sensitivity**	**Specificity**
Linear	97.4%	98.7%	96.3%
Gaussian	50.6%	50.3%	100.0%
Polynomial	97.4%	100.0%	95.1%
**Inceptionv3 [24]**			
**Kernel**	**Accuracy**	**Sensitivity**	**Specificity**
Linear	95.5%	93.8%	97.3%
Gaussian	50.6%	50.3%	100.0%
Polynomial	63.5%	100.0%	57.8%
**ResNet-50 [22]**			
**Kernel**	**Accuracy**	**Sensitivity**	**Specificity**
Linear	96.8%	98.7%	95.1%
Gaussian	50.6%	50.3%	100.0%
Polynomial	95.5%	100.0%	91.8%
**GoogLeNet [25]**			
**Kernel**	**Accuracy**	**Sensitivity**	**Specificity**
Linear	95.5%	96.1%	944.9%
Gaussian	50.6%	50.3%	100.0%
Polynomial	96.8%	98.7%	95.1%
**VGG-19 [23]**			
**Kernel**	**Accuracy**	**Sensitivity**	**Specificity**
Linear	96.8%	97.4%	96.2%
Gaussian	50.6%	50.3%	100.0%
Polynomial	93.6%	97.2%	90.5%

**Table 3:** Classification results of audio descriptors.

**MFCC [20]**			
**Kernel**	**Accuracy**	**Sensitivity**	**Specificity**
Linear	81.7%	85.0%	75.0%
Gaussian	98.3%	97.5%	100.0%
Polynomial	63.3%	94.7%	48.8%
**LPCC [19]**			
**Kernel**	**Accuracy**	**Sensitivity**	**Specificity**
Linear	65.0%	100.0%	65.0%
Gaussain	63.3%	97.4%	64.4%
Polynomial	83.3%	86.7%	82.2%

**Table 4:** Comparison of proposed method with existing methods.

Ref No.	Audio Data	Image Data	Sample Approach	Accuracy
[29]	√	-	Deep Belief Network	88.0%
[36]	√	-	Correlation	70%
[38]	√	-	HMM	81.3%
[47]	√	-	SVM with Genetic Algorithm	95.0%
**This Paper**	**√**	**-**	**MFCC**	**98.3%**
[48]	-	√	ResNet-50	96.8%
[49]	-	√	FD-HOG	82.7%
[50]	-	√	LBP and HOG	62.2% and 82.7%
**This Paper**	**-**	**√**	**AlexNet**	**97.4%**
[26]	√	√	HOG and MFCC	82.7% and 98.3%
**This Paper**	**√**	**√**	**AlexNet and MFCC**	**98.5%**

## References

[1] Rice A.B. Drone technology as applied to the cement industry. Proceedings of the 2016 IEEE-IAS/PCA Cement Industry Technical Conference.

[2] Albornoz C., Giraldo L.F. Trajectory design for efficient crop irrigation with a UAV. Proceedings of the 2017 IEEE 3rd Colombian Conference on Automatic Control (CCAC).

[3] Spoorthi S., Shadaksharappa B., Suraj S., Manasa V.K. Freyr drone: Pesticide/fertilizers spraying drone—An agricultural approach. Proceedings of the 2017 2nd International Conference on Computing and Communications Technologies (ICCCT).

[4] Al Shamsi M., Al Shamsi M., Al Dhaheri R., Al Shamsi R., Al Kaabi S., Al Younes Y. Foggy drone: Application to a hexarotor UAV. Proceedings of the 2018 Advances in Science and Engineering Technology International Conferences (ASET).

[5] L-Hourani A.A., Chandrasekharan S., Kaandorp G., Glenn W., Jamalipour A., Kandeepan S. (2016). Coverageand rate analysis of aerial base stations. IEEE Trans. Aerosp. Electron. Syst..

[6] He D., Chan S., Guizani M. (2017). Drone-Assisted Public Safety Networks: The Security Aspect. IEEE Commun. Mag..

[7] Vattapparamban E., Güvenç I., Yurekli A.Ì., Akkaya K., Uluagaç S. Drones for smart cities: Issues in cybersecurity, privacy, and public safety. Proceedings of the 2016 International Wireless Communications and Mobile Computing Conference (IWCMC).

[8] Gharibi M., Boutaba R., Waslander S.L. (2016). Internet of Drones. IEEE Access.

[9] Brust M.R., Danoy G., Bouvry P., Gashi D., Pathak H., Gonçalves M.P. Defending Against Intrusion of Malicious UAVs with Networked UAV Defense Swarms. Proceedings of the 2017 IEEE 42nd Conference on Local Computer Networks Workshops (LCN Workshops).

[10] Ding G., Wu Q., Zhang L., Lin Y., Tsiftsis T.A., Yao Y. (2018). An Amateur Drone Surveillance System Based on the Cognitive Internet of Things. IEEE Commun. Mag..

[11] Anwar M.Z., Kaleem Z., Jamalipour A. (2019). Machine Learning Inspired Sound-Based Amateur Drone Detection for Public Safety Applications. IEEE Trans. Veh. Technol..

[12] Saleh S.A., Azam S., Yeo K.C., Shanmugam B., Kannoorpatti K. An improved face recognition method using Local Binary Pattern method. Proceedings of the 2017 11th International Conference on Intelligent Systems and Control (ISCO).

[13] Li Y., Su G. Simplified histograms of oriented gradient features extraction algorithm for the hardware implementation. Proceedings of the 2015 International Conference on Computers, Communications, and Systems (ICCCS).

[14] Song T., Li H., Meng F., Wu Q., Cai J. (2018). LETRIST: Locally Encoded Transform Feature Histogram for Rotation-Invariant Texture Classification. IEEE Trans. Circuits Syst. Video Technol..

[15] Costianes P.J., Plock J.B. Gray-level co-occurrence matrices as features in edge enhanced images. Proceedings of the 2010 IEEE 39th Applied Imagery Pattern Recognition Workshop (AIPR).

[16] Wu X., Sun J. (2017). Joint-scale LBP: A new feature descriptor for texture classification. Vis. Comput..

[17] Murala S., Maheshwari R.P., Balasubramanian R. (2012). Local Tetra Patterns: A New Feature Descriptor for Content-Based Image Retrieval. IEEE Trans. Image Process..

[18] Nguyen D.T., Zong Z., Ogunbona P., Li W. Object detection using Non-Redundant Local Binary Patterns. Proceedings of the 2010 IEEE International Conference on Image Processing.

[19] Gupta H., Gupta D. LPC and LPCC method of feature extraction in Speech Recognition System. Proceedings of the 2016 6th International Conference—Cloud System and Big Data Engineering (Confluence).

[20] Kumar A., Rout S.S., Goel V. Speech mel frequency cepstral coefficient feature classification using multi level support vector machine. Proceedings of the 2017 4th IEEE Uttar Pradesh Section International Conference on Electrical, Computer and Electronics (UPCON).

[21] Sun J., Cai X., Sun F., Zhang J. Scene image classification method based on Alex-Net model. Proceedings of the 2016 3rd International Conference on Informative and Cybernetics for Computational Social Systems (ICCSS).

[22] Xiao X., Wan W. Human pose estimation via improved ResNet50. Proceedings of the 4th International Conference on Smart and Sustainable City (ICSSC 2017).

[23] Kwasigroch A., Mikolajczyk A., Grochowski M. Deep neural networks approach to skin lesions classification -- A comparative analysis. Proceedings of the 2017 22nd International Conference on Methods and Models in Automation and Robotics (MMAR).

[24] Ali M.A., el Munim H.E.A., Yousef A.H., Hammad S. A Deep Learning Approach for Vehicle Detection. Proceedings of the 2018 13th International Conference on Computer Engineering and Systems (ICCES).

[25] Salavati P., Mohammadi H.M. Obstacle Detection Using GoogleNet. Proceedings of the 2018 8th International Conference on Computer and Knowledge Engineering (ICCKE).

[26] Liu H., Wei Z., Chen Y., Pan J., Lin L., Ren Y. Drone detection based on an audio-assisted camera array. Proceedings of the 2017 IEEE Third International Conference on Multimedia Big Data (BigMM).

[27] Lee S.J., Jung J.H., Park B. Possibility verification of drone detection radar based on pseudo random binary sequence. Proceedings of the 2016 International SoC Design Conference (ISOCC).

[28] Drozdowicz J., Wielgo M., Samczynski P., Kulpa K., Krzonkalla J., Mordzonek M., Byrl M., Jakielaszek Z. 35 GHz FMCW drone detection system. Proceedings of the 2016 17th International Radar Symposium (IRS).

[29] Mendis G.J., Randeny T., Wei J., Madanayake A. Deep learning based doppler radar for micro UAS detection and classification. Proceedings of the MILCOM 2016—2016 IEEE Military Communications Conference.

[30] Tang F., Mao B., Fadlullah Z.M., Kato N., Akashi O., Inoue T., Mizutani K. (2018). On removing routing protocol from future wireless networks: A real-time deep learning approach for intelligent traffic control. IEEE Wireless Commun..

[31] Fawad M.J., Khan M.A., Riaz H., Shahid M.S., Khan Y., Amin J., Loo H., Tenhunen H. (2019). Texture Representation through Overlapped Multioriented Tri-scale Local Binary Pattern. IEEE Access.

[32] Saeed A., Fawad, Khan M.J., Riaz M.A., Shahid H., Khan M.S., Amin Y., Loo J., Tenhunen H. (2019). Robustness-Driven Hybrid Descriptor for Noise-Deterrent Texture Classification. IEEE Access.

[33] Fawad M.J., Khan M., Rahman Y., Amin H. (2019). Tenhunen Low-Rank Multi-Channel Features for Robust Visual Object Tracking. Symmetry.

[34] Rydén H., Redhwan S.B., Lin X. (2018). Rogue drone detection: A machine learning approach. arXiv.

[35] Kim J., Park C., Ahn J., Ko Y., Park J., Gallagher J.C. Real-time UAV sound detection and analysis system. Proceedings of the 2017 IEEE Sensors Applications Symposium (SAS).

[36] Mezei J., Molnar A. Drone sound detection by correlation. Proceedings of the 2016 IEEE 11th International Symposium on Applied Computational Intelligence and Informatics (SACI).

[37] Muller T. Robust drone detection for day/night counter-UAV with static VIS and SWIR cameras. Proceedings of the Ground/Air Multisensor Interoperability, Integration, Networking Persistent ISR VIII.

[38] Shi L., Ahmad I., He Y., Chang K. (2018). Hidden Markov model-based drone sound recognition using MFCC technique in practical noisy environments. J. Commun. Netw..

[39] Rai P., Golchha V., Srivastava A., Vyas G., Mishra S. An automatic classification of bird species using audio feature extraction and support vector machines. Proceedings of the 2016 International Conference on Inventive Computation Technologies (ICICT).

[40] Parikh K.S., Shah T.P. (2016). Support vector machine--a large margin classifier to diagnose skin illnesses. Procedia Technol..

[41] Tanwar R., Malhotrab S. (2017). Scope of Support Vector Machine in Steganography. IOP Conference Series: Materials Science and Engineering.

[42] UAVs Detection Using Images with Handcrafted Descriptors MATLAB Code. https://github.com/SonainJamil/UAV-Detection-using-images-part-01.git.

[43] UAVs Detection Using Images with D-CNN Models MATLAB Code. https://github.com/SonainJamil/UAV-Detection-using-images.git.

[44] UAVs Detection Using Audios MATLAB Code. https://github.com/SonainJamil/UAV-Detection-using-Audio.git.

[45] Malicious UAVs Detection Dataset. https://www.kaggle.com/sonain/malicious-uavs-detection.

[46] UAVs Detection Paper Code. https://github.com/SonainJamil/Malicious-UAV-Detection-Code.git.

[47] He Y., Ahmad I., Shi L., Chang K. (2019). SVM-based drone sound recognition using the combination of HLA and WPT techniques in practical noisy environment. KSII Trans. Internet Inf. Syst..

[48] Abbasi K., Batool A., Asghar M.A., Saeed A., Khan M.J., ur Rehman M. A Vision-Based Amateur Drone Detection Algorithm for Public Safety Applications. Proceedings of the 2019 UK/ China Emerging Technologies (UCET).

[49] Wang Z., Qi L., Tie Y., Ding Y., Bai Y. Drone Detection Based on FD-HOG Descriptor. Proceedings of the 2018 International Conference on Cyber-Enabled Distributed Computing and Knowledge Discovery (CyberC).

[50] Gökce F., Ücoluk G., Sahin E., Kalkan S. (2015). Vision-based detection and distance estimation of micro unmanned aerial vehicles. Sensors.

